# Role of salivary MMP-9 in OSCC detection and diagnosis: A comprehensive clinical assessment

**DOI:** 10.6026/973206300211680

**Published:** 2025-06-30

**Authors:** Pranav Manek, Vijayta Singh, Gajendra Singh Sisodia, Indu Tomar, Dax Abraham, Parul Agarwal

**Affiliations:** 1 Department of Oral Medicine and Radiology, Pacific Dental College and Research Centre, Udaipur, Rajasthan, India; 2 Department of Pathology, Maharaja Suhel Dev Autonomous State Medical College & Mahrishi Balark Hospitals, Bahraich, Uttar Pradesh, India; 3 Department of Community Medicine, Pacific Medical College & Hospital, Udaipur, Rajasthan, India; 4 Department of Biochemistry, Research Scholar (PhD), Department of Biosciences, Manipal University, Jaipur, Rajasthan, India; 5 Department of Conservative Dentistry and Endodontics, Manav Rachna Dental College, School of Dental Sciences, Manav Rachna International Institute of Research And Studies, Faridabad, Haryana, India; 6 Department of Oral Medicine and Dental Radiology, K D Dental College and hospital, Mathura, Uttar Pradesh, India

**Keywords:** matrix metalloproteinase, MMP-9, Oral squamous cell carcinoma, salivary biomarker, saliva

## Abstract

An Enzyme-Linked Immunosorbent Assay (ELISA) to measure the salivary Matrix metalloproteinase-9 (MMP-9) levels among squamous cell carcinoma patients was used. Thirty of the sixty participants in the research had a verified diagnosis of oral SCC, whereas the remaining twenty were healthy controls. Salivary MMP-9 levels were extracted by centrifugation and measured using ELISA. The results were compared between oral SCC patients and controls. Participants with oral squamous cell carcinoma had considerably greater mean salivary MMP-9 levels than healthy subjects (p<0.05). Thus, individuals with oral squamous cell carcinoma have average salivary MMP-9 levels, and those with poorly differentiated oral squamous cell carcinoma have even higher levels.

## Background:

Cancer of the oral cavity and lip together constitute the sixth most common cancer globally where 90% of the cases are of OSCC (oral squamous cell carcinoma) alone. Tumor markers in the saliva have evolved as a new diagnostic modality in the detection of oral carcinoma [1]. In the saliva of the subjects with carcinoma, a higher number of enzymes, proteins and other chemicals can be gathered and assessed. Collection and sampling of the saliva are a relatively simple procedure and also saliva is easily accessible bio fluid in comparison to blood sampling and tissue biopsies [2]. Changes in the levels of MMPs (Matrix Metalloproteinase) are usually related to the ultimate clinical disease outcomes in humans. MMPs can degrade the basement membrane matrix, ECM (extracellular matrix) and their related components. MMP-9 is a gelatinase that plays a vital role in the process of tumorigenesis [3]. Therefore, it is of interest to assess the levels of salivary MMP-9 in subjects with oral squamous cell carcinoma assessed using ELISA (Enzyme-Linked Immunosorbent Assay), present study was performed.

## Materials and Methods:

The present comparative observational study was done at Department of Pathology after the clearance was taken by the concerned Institutional Ethical committee. Verbal and written informed consent was taken from all the subjects before study participation. The study included 60 subjects that were equally distributed to the two groups of 15 subjects each. Group I had confirmed histopathological and clinical diagnosis of OSCC and Group II included control group. Saliva samples were collected from all the subjects by instructed to open their mouth slightly to allow the draining of the saliva to the container to collect 1.5ml of unstimulated whole saliva into a sterile centrifuge tube. After collection of the saliva, it was centrifuged immediately and the supernatant was collected and frozen at -80 °C till assay and further processing. Samples and standards were added to appropriate microtiter plate wells followed by the addition of a biotin-conjugated antibody specific to MMP-9. This conjugated enzyme avidin and biotin-conjugated antibody specific to MMP-9 depict the colour change. This substrate enzyme reaction termination was done by adding sulphuric acid solution and spectrophotometric measurement of color change was evaluated at 450nm wavelength. MMP-9 concentration in the samples was assessed in ng/ml by comparison of the OD (optical density) values of the samples. The data gathered were statistically analysed using SPSS software version 24.0 for assessment of descriptive measures, Student t-test, ANOVA, Fisher's exact test, Mann-Whitney U test and Chi-square test. The results were expressed as mean and standard deviation and frequency and percentages. The p-value of <0.05 was considered.

## Results:

The mean age of the study subjects in OSCC and control groups was 64 ± 3.8 and 60 ± 3.3 years which was statistically comparable. The majority of the study subjects were aged 51-60 and >70 years in the OSCC group with 33.3% (n=10) subjects each followed by 26.6% (n=8) subjects in 61-70 and 6.66% (n=2) subjects from 31-40 years respectively. In the control group, the majority of subjects were from 61-70 years with 53.3% (n=16) subjects followed by 40 (n=12) subjects in 51-60 years and 6.66% (n=2) subjects from >70 years respectively (Table 1 - see PDF). The study results showed that on comparison of MMP-9 levels in subjects with oral squamous cell carcinoma from Group I mean salivary MMP-9 levels were 48.6 ± 5.5 ng/ml which was significantly higher when compared to salivary MMP-9 levels in Group II (control) group subjects where it was 16.9 ± 4.6 ng/dl with p-value of <0.01. However, a non-significant difference and statistically comparable values were seen for serum MMP-9 levels in well-differentiated, moderately differentiated and poorly differentiated oral squamous cell carcinoma with p=0.07 (Table 2 - see PDF).

## Discussion:

The mean age of the study subjects in OSCC and control groups was 64 ± 3.8 and 60 ± 3.3 years which was statistically comparable. The majority of the study subjects were aged 51-60 and >70 years in the OSCC group with 33.3% (n=10) subjects each followed by 26.6% (n=8) subjects in 61-70 and 6.66% (n=2) subjects from 31-40 years respectively. In the control group, the majority of subjects were 61-70 years with 53.3% (n=16) subjects followed by 40 (n=12) subjects in 51-60 years and 6.66% (n=2) subjects from >70 years respectively. These data were comparable to the previous studies of Brocklehurst *et al.* [4] in 2013. The study results showed that among the 30 subjects included in Group I, 91% of subjects reported a positive history of tobacco use, whereas, 9% of subjects reported no history of any deleterious habit. In Group I, OSCC group, the majority of the lesions were in the buccal mucosa with 52% lesions followed by the tongue, vestibule, gingiva, alveolar ridge, lips and floor of the mouth with 23%, 8%, 5%, 6%, 3% and 3% lesions respectively. These results were consistent with the studies of Peisker *et al.* [5] in 2017 and Dalirsani *et al.* [6] in 2019. It was seen that group I with 30 subjects having oral squamous cell carcinoma were assessed histopathologically to judge the differentiation. It was seen that there were 10 lesions each categorized as well differentiated, moderately differentiated and poorly differentiated lesions where 58.8%, 26.5% and 14.7% lesions each were well-differentiated, moderately differentiated and poorly differentiated respectively. These findings were in agreement with the results of Kawas *et al.* [7] in 2012 and Ruokolainen *et al.* [8] in 2004. A non-significant difference and statistically comparable values were seen for serum MMP-9 levels in well-differentiated, moderately differentiated and poorly differentiated oral squamous cell carcinoma with p=0.07. These results were in line with the findings of Katayama *et al.* [9] in 2004 and Shpitzer *et al.* [10] in 2007.

## Conclusion:

We show that mean salivary MMP-9 levels are seen in subjects with oral squamous cell carcinoma with further higher levels in subjects with poorly differentiated oral squamous cell carcinoma. Thus, the potential role of MMP-9 as a prognostic biomarker in oral squamous cell carcinoma is shown. However, further longitudinal studies with larger sample sizes and longer monitoring periods are needed to reach a definitive conclusion.
